# Practical Aspects of Acne Scar Management: ASAP 2024

**DOI:** 10.7759/cureus.55897

**Published:** 2024-03-10

**Authors:** Sushil Tahiliani, Venkatraman Mysore, Anil Ganjoo, Satish Udare, SC Rajendran, Raghunatha Reddy, V S Rathore, Satish Bhatia, Sachin Verma, Vaishali Katke, Chetan Y Patil

**Affiliations:** 1 Dermatology, Dr. Sushil Tahiliani Clinic, Mumbai, IND; 2 Dermatology, P. D. Hinduja Hospital and Medical Research Centre, Mumbai, IND; 3 Dermatology, The Venkat Centre for Skin and Plastic Surgery, Bengaluru, IND; 4 Dermatology, Skinnovation Clinics, New Delhi, IND; 5 Dermatology, Dr. Udare Skin and Hair Clinic, Navi Mumbai, IND; 6 Dermatology, Cosmetic Skin Care Clinic, Bengaluru, IND; 7 Dermatology, Roots Institute of Dermatological Sciences, Bengaluru, IND; 8 Plastic Surgery, Kaayakalp Clinic, Kolkata, IND; 9 Dermatology, Indian Cancer Society, Mumbai, IND; 10 Dermatology, Skinvita Clinic, Kolkata, IND; 11 Medical Affairs, A. Menarini India Pvt Ltd., Mumbai, IND

**Keywords:** acne scar treatment, scar improvement, atrophic scar, acne scar, acne vulgaris

## Abstract

Acne scars are one of the most common complications of acne. They can significantly affect the patient's quality of life. Often, several types of atrophic acne scars are observed simultaneously; therefore, consideration must be given to the type of scar while choosing the treatment modality. Effective treatment is not only important to prevent and improve acne scars but also crucial in preventing psychosocial effects. Treatment of acne scars requires an algorithmic approach that targets each component of the scars, and combination therapy on a patient-specific basis may offer the best chance for significant improvement. The goal of the current article is to discuss the practical aspects of management of atrophic acne scars using the vast modalities of treatment available. The panel of dermatologists and plastic surgeons, each one with at least 20 years of experience in acne scar treatment, participated in a series of ‘Practical Aspects of Acne Scar Management' (ASAP) meetings: ASAP 2024. ASAP meetings were organized by "Scar Forum India" from March 2023 to July 2023 in four Indian cities (Mumbai, Delhi, Bengaluru, and Kolkata), each one for a duration of at least three hours. During these meetings and discussions, panelists reviewed and discussed the acne scar-related literature, their clinical experience in its management, available treatment options, along with recent advances. Consequently, a summary of the discussion and practical approach for the management of acne scars is developed. It was concluded that, though there is no specific guideline available to optimize acne scar management despite the multitude of treatment options, the best results can be achieved through the synergy of multiple treatment modalities and using the algorithmic approach.

## Introduction and background

Acne is one of the most prevalent skin conditions, often occurring during adolescence, affecting up to 80% of this age group. It commonly appears on the face, chest, and back (an area that has a high presence of sebaceous glands) [1-3]. Acne scars are a usual consequence of acne, which can occur in up to 95% of patients, with facial acne equally common in men and women [4]. The exact prevalence of acne scarring in the general population is mostly unknown [3]. In a study conducted on more than 6,000 Indian acne patients, the prevalence of acne scars was noted in 29% of patients. Another Indian study documented acne scars in about 40% of the total study patients [4].

Post-acne scarring causes an unwanted cosmetic appearance, affecting the person’s mental health, social functioning, and general well-being, resulting in a reduced quality of life [4,5]. Studies have emphasized the significant psychosocial impact of atrophic acne scars on patients in the form of embarrassment and self-consciousness. Patients with acne scarring have demonstrated a notable impact on their quality of life, even with mild acne scars, and this impact has grown as the scar's severity increased [6,7]. Therefore, opting for effective treatment modalities to prevent and ameliorate acne scarring is crucial in preventing psychosocial effects.

A panel of dermatologists and plastic surgeons, each with at least 20 years of experience in acne scar treatment, participated in a series of four ‘Practical Aspects of Acne Scar Management’ (ASAP) meetings: ASAP 2024. ASAP meetings were organized by ‘Scar Forum India’ from March 2023 to July 2023 in four Indian cities (Mumbai, Delhi, Bengaluru, and Kolkata), each for a duration of at least three hours. During these meetings and discussions, they reviewed and discussed the literature, clinical experience on acne scar, its pathophysiology, management, available topical treatment, energy-based and non-energy-based therapies for acne scar management, along with the recent advances of the same. The outcome of these meetings was captured and then developed into a practical approach for the management of acne scars with their extensive clinical expertise and experience.

## Review

Most scars result from impairment of the acne healing process. The two main types of acne scars, atrophic and hypertrophic, can be distinguished based on whether there is an overall reduction or buildup of collagen [2]. About 90% of people with acne scars have atrophic acne scars, while very few have hypertrophic scars and keloids. The pathogenesis of atrophic acne scars is mainly related to the levels of inflammatory mediators, degradation of collagen, and subcutaneous fat [2,3]. People who are not prone to acne scarring show a strong initial inflammatory response to acne that is effective at quickly repairing tissue. This inflammatory response returns to normal levels as the tissue is repaired. On the other hand, acne-prone individuals (Table 1) exhibit inadequate and delayed or prolonged inflammation that is not efficient for tissue repair. This delayed and/or prolonged inflammation is considered to be a primary pathophysiology behind acne scarring [4,8].

**Table 1:** Risk factors for acne scarring

Sr. no.	Risk factors	Description
1	Inflammatory acne	These often include acne cysts and nodules. This type of acne tends to penetrate deep into the skin, which damages the skin
2	Delayed or no treatment of inflammatory acne	Longer a person has inflammatory acne, the greater the risk of scarring
3	Picks, squeezes, or pops acne	This increases inflammation, which increases the risk of scarring
4	Gender	Male gender might be at higher risk for acne scars. This is more likely because men suffer from severe acne than women, which might be associated with high androgen levels and special sebaceous gland
5	Genetics factors	Positive family history of acne

Three types of atrophic acne scars can be distinguished: rolling, boxcar, and ice pick scars (Table 2) [2]. Classification of scar types is crucial since it influences the treatment options available. Ice-pick scars usually extend deep into the dermis, making them difficult to treat with conventional skin-resurfacing treatments. In the case of rolling scars, treatment at the subdermal level is necessary because they are wider and have fibrous anchoring to the subcutis. Shallower boxcar scars respond better to skin resurfacing procedures; however, deeper boxcar scars are more resistant to such superficial treatments. Another important consideration in the management of acne scars is their severity, which can also be used to classify scars (Table 3) [9,10].

**Table 2:** Acne scar subtypes

Acne scars subtype	Clinical features
Ice pick	Ice pick scars are narrow (<2 mm), deep, and sharply demarcated tracts that extend vertically to the deep dermis or subcutaneous tissue (forming a “V” shape)
Rolling	Rolling scars may reach ≥5 mm in diameter. They have a rolling or undulating appearance (forming “M” shape) that occurs from fibrous tethering of the dermis to the subcutis
Boxcar	Boxcar scars are oval depressions with sharply demarcated vertical edges. They are wider at the surface than ice pick scars and do not taper to a point at the base (forming “U” shape)

**Table 3:** Acne scar severity grading scale

Grade	Level of lesion	Clinical features
1	Macular	Macular erythematous, hyperpigmented, or hypopigmented flat marks
2	Mild	Mild atrophic or hypertrophic scarring that may not be obvious at social distances of 50 cm or greater and easily covered by makeup or beard hair in men
3	Moderate	Moderate atrophic or hypertrophic scarring that is obvious at social distances of 50 cm or greater and is not covered easily by makeup or beard hair in men, but is still able to be flattened by manual stretching of the skin
4	Severe	Severe atrophic or hypertrophic scarring not flattened by manual stretching of the skin

Since different atrophic scar types are frequently seen at the same time and can be challenging to distinguish, several authors have proposed a number of additional classifications and scales. Based on the kind and quantity of scars, Goodman and Baron created a quantitative tool for assessing global acne scarring (Table 4) [2,10]. Dreno et al.'s ECCA (Echelle d'Evaluation Clinique des Cicatrices d'acné) is another quantitative scale that has been proposed for facial acne scarring. The ECCA classification is based on the total of all scar types and their numerical extent, with scar types that are notably disfiguring receiving a higher weight [2,11].

**Table 4:** Goodman and Baron scale of acne scar [2,10] Multiplication factor: 1, Number of lesions: 1–10; 2, Number of lesions: 11–20; 3, Number of lesions: > 20.

Acne scar type	Acne scar description	Score
Milder scarring	(1) Macular erythematous or pigmented and (2) mildly atrophic dish-like	1 point each
Moderate scarring	(1) Moderately atrophic dish-like, (2) punched out with shallow bases and small scars (< 5 mm), and (3) shallow but broad atrophic areas	2 points each
Severe scarring	(1) Punched out with deep but normal bases and small scars (<5 mm), (2) punched out with deep abnormal bases and small scars (<5 mm), (3) linear or troughed dermal scarring, and (4) deep broad atrophic areas	3 points each
Hyperplastic	Papular scars	2 points
	Keloidal/hypertrophic scars	6 points

The best way to treat acne scarring is prevention by treating active acne as early as possible. After scarring has occurred, the patient's needs should be taken into account when designing a treatment plan. Regardless of the strategy, patients need to be guided toward realistic expectations and offered realistic options [12]. Treatment options that are commonly used can be broadly classified as energy-based or non-energy-based modalities (Table 5). A thorough consultation should always precede any safe and efficient treatment for acne scars [2,12], including setting expectations regarding realistic outcomes and downtime in addition to the examination of the skin and scars [12,13]. This also helps in a better understanding of patient needs, which ultimately aids in better treatment planning for optimal results.

**Table 5:** Classification of different treatment modalities in scar management

Pharmacological topical	Non-surgical procedures	Energy-based devices
Chemical peels	Subcision	Ablative: CO_2_, Nd:YAG, YSGG
Topical peptides	Punch excision/punch elevation/punch replacement grafting	Nonablative: Nd:YAG, PDL, Er-doped, Er:glass
	Dermabrasion/microdermabrasion	Radiofrequency
	Needling	
	Soft tissue augmentation (dermal fillers)	
	Platelet-rich plasma	
	Stem cell	

Chemical peels

Phenol was the first chemical product to be used topically to treat acne scars, according to historical accounts from the 1950s [12]. Today, aestheticians frequently employ them as a popular technique to treat acne scars and rejuvenate skin. When applied to the skin's dermal or epidermal layers, chemical peels, which are acids in nature, produce controlled microinjuries [2]. This results in new collagen synthesis and exfoliation during the extracellular matrix (ECM) remodeling process [12]. They have strong keratolytic effects that stimulate new collagen synthesis, increase squamation and epidermolysis, and reduce corneocyte adhesion. Chemical peels are available in different grades and of different types (Table 6) [12-15]. Depending on their potency, they can be used to treat a range of skin issues, including photoaging, hyperpigmentation, acne, and acne scars [12]. It is also advised to use topical tretinoin for two weeks prior to and following the procedure in order to exfoliate the skin. This accelerates the process of re-epithelialization, improving both the rate of recovery and the depth of the deep peeling agent [2,16]. Patients frequently choose peels because they are comparatively non-invasive and can simultaneously improve the texture, tone, and pigmentation of the skin [15,16].

**Table 6:** Classification of chemical peels GA, glycolic acid; TCA, trichloroacetic acid.

Type (depth of penetration)	Histologic level	Peeling agents	Clinical implication
Very superficial	Destruction of the stratum corneum without creating a wound below the stratum granulosum	GA, 30–50%, applied briefly (1–2 min); Jessner’s solution, applied in 1 to 3 coats; TCA 10%, applied in 1 coat	Very superficial acne scars better suited for improving post-inflammatory skin pigmentation
Superficial	Destruction of part or all of the epidermis, anywhere from the stratum granulosum to the basal cell layer	GA, 50–70%, applied for a variable time (2–20 min); Jessner’s solution, applied in 4 to 10 coats; TCA, 10–30%	Superficial acne scars and may be best suited for improving post-inflammatory skin pigmentation
Medium	Destruction of the epidermis and part or all of the papillary dermis	GA 70%, applied for a variable time (3–30 min); TCA, 35–50%; Augmented TCA (CO_2_ plus TCA 35%; Jessner’s solution plus TCA 35%; GA 70% plus TCA 35%)	Effective for deep atrophic scars, but carry a higher risk of complications, such as milia, post-procedure pigmentary changes, and secondary infection
Deep	Destruction of the epidermis and papillary dermis, extending into the reticular dermis	Phenol 88%; TCA, >50%; Baker-Gordon phenol formula	Phenol peels are currently used infrequently because of associated cardiac toxicity related to systemic absorption. Effective for deep atrophic scars like ice pick scars, but carry a higher risk of complications, such as milia, post-procedure pigmentary changes, and secondary infection

Scarring and post-inflammatory pigmentation (PIH) are frequent side effects of chemical peels [12]. Stronger, deeper peels carry a higher risk. Darker skin types (Fitzpatrick skin types IV-VI) and noncompliance with aftercare instructions (avoidance of sun exposure, use of sunscreen, etc.) are associated with an increased risk of PIH [2,16]. The majority of research, however, indicates that PIH is transient and that topical depigmenting agents can be used both before and after treatment to prevent it. Hypopigmentation, erythema, pain, and irritation are other frequent side effects of chemical peels that can happen following the treatment, but they are usually transient and resolve spontaneously without any need for further treatment [15,16].

Chemical reconstruction of skin scarring (CROSS), a novel application method, has been applied recently to target specific scars with focused doses of deep peeling agents, lowering the risk of scarring and dyspigmentation that comes with full-face application. Trichloroacetic acid (TCA)-CROSS applies a high concentration of TCA locally and repeatedly to the scars using dull point wooden applicators. This method can be used without the need for sedation or local anesthesia [2,15,16]. Studies have demonstrated that high-concentration TCA (50-100%) can be used with the CROSS method to treat atrophic scars locally and effectively with few side effects. Furthermore, a few studies have indicated that a 50% TCA concentration produces outcomes comparable to those of 90% TCA with significantly fewer negative effects. TCA is applied to the scar for a brief period of time, and sessions can be repeated three to four times at one-month intervals. Following the procedure, prescriptions for emollients and high SPF sunscreen are required. This approach helps in minimizing the risk of scarring and hypopigmentation [2].

Topical peptides

Topical peptides have emerged as a promising new adjuvant modality of routine treatment. Clinical studies have demonstrated the beneficial effects of topical peptides when used as monotherapy or as an adjunctive treatment for acne scarring [4]. It has been shown in the studies that topical peptides following invasive resurfacing procedures promote healing and reduce downtime, leading to better patient outcomes and improved patient satisfaction [17-20].

One of the newest topical formulations available for the treatment of post-acne scars in India is a combination of Kollaren and Exo-T, which is a combination of biomimetic tripeptide and purified exopolysaccharide [4]. Kollaren, a tripeptide, promotes the synthesis of ECM proteins like collagen (Types I and III), laminin, fibronectin, and elastin. Meanwhile, Exo-T contributes to the healing process by stimulating desquamation and cell differentiation [4,21,22]. A study conducted by Kandhari et al. on Indian patients using tripeptide reported significant improvement in acne scarring in the majority of participants, about 80% in three months [4]. Apart from scar gradings, there was also a significant reduction in pigmentation. Based on available evidence, topical peptides can be a good home-based treatment option as an adjunctive to other acne scar modalities and an option to use immediately after post-acne treatment to reduce the chances of scarring and pigmentation [4].

Subcision

First described in the early 1990s, subcision is an easy but effective treatment for superficial atrophic acne scars [23]. To perform this procedure, an 18-G nokor or tri-beveled hypodermic needle is inserted into the dermis below the scar horizontally and passed in multiple directions parallel to the skin. Subcision is effective for the rolling type of acne scars, while deep scars like boxcar and ice pick scars are less treatable [12]. Scar improvement occurs due to the freeing of fibrotic tissue beneath the scars and the synthesis of new connective tissue in the area [23]. It typically requires several treatment sessions to achieve the best results. Among the many benefits of subcision are its ease of execution, low cost, quick recovery, suitability for a range of skin types (I-IV), lack of significant side effects, and quick, persistent results [24]. Common complications of subcision include bruising, bleeding, transient discoloration, infection, hypertrophic scars, and the risk of acne aggravation if acne sinus tracts are disrupted while performing the procedure. Subcision can be used alone or in combination with dermal fillers, which then can occupy the space created by subcision rather than depending purely on the natural healing process to fill the space created [5,12].

Punch excision

Punch excision, sometimes referred to as elliptical excision, is performed at the subcutaneous level. It is a useful technique for eliminating deep scars like deep boxcar or ice pick acne scars [12]. Using a punch biopsy tool, deep atrophic scar tissue is removed up to the subcutaneous layer, and then the exposed area is closed with a suture. Usually, the newly formed scar following excision is not as noticeable as the previous acne scar. To prevent excessive traction on the skin, scars should be spaced a minimum of 4-5 mm apart. Alternatively, at least four to five weeks should pass between procedures to prevent side effects. For the best cosmetic result, the procedure is advised for scars larger than three mm and can be used in conjunction with later procedures like dermabrasion or laser skin resurfacing [12,23].

Punch elevation

Punch elevation is a highly efficient method for treating both shallow and deep boxcar scars. This method comprises a combination of punch excision and grafting. First, the scar is cautiously excised using a punch biopsy tool, and then, the tissue is gently lifted marginally above the surface of the skin. To secure the elevated tissue in place, sutures or sterile strips are used [12]. As the wound heals, the graft settles and retracts slightly toward the surrounding skin, significantly optimizing the scar appearance. Additionally, punch elevation can be followed by laser treatment within two months [12,23]. A recent study suggests that combining punch elevation with fractional CO_2_ laser yields superior outcomes in acne scars when compared to fractional CO_2_ laser alone [12,25].

Punch replacement grafting

This technique is very useful for deep or sharp-walled ice pick scars. It can produce the best results for challenging, sharply defined scars, but it is a laborious method that frequently necessitates multiple replacement grafts in a single session. Slightly larger full-thickness skin grafts are used, preferably from the postauricular area. Certain grafts will heal at the skin's surface level, while others will heal at an elevated level. The doctor's skill is a major factor in this technique's success. Both graft failure and a pronouncedly unevenly matched appearance are possible outcomes after the procedure. In most cases, it yields long-term results [5,12].

Dermabrasion and microdermabrasion

In the early 1900s, dermatologist Kromayer played a significant role in the advancement of dermabrasion methods [26]. Over the past few decades, numerous advancements in procedure, tools, and anesthesia have been made. Dermabrasion requires a high level of skill, and there is a significant variation in clinical results between operators [26]. These techniques mechanically ablate the skin to encourage tissue re-epithelialization, leading to skin resurfacing [2]. Although the skin is physically abraded during both dermabrasion and microdermabrasion, the instruments used and their technical executions differ [2]. Dermabrasion stimulates the remodeling of the skin's structural proteins by completely removing the epidermis and reaching the level of the reticular dermis. On the other hand, microdermabrasion is a superficial form of dermabrasion that eliminates the epidermis, triggering the body's natural exfoliation process. Both methods result in scar appearance improvements that are clinically significant [2,26]. Microdermabrasion has less serious side effects than dermabrasion, is painless, and can be repeated frequently. It also doesn't require anesthesia. However, microdermabrasion is not useful for treating deep scars. It is a less expensive option compared to laser skin resurfacing therapy [1,2,26].

Needling and microneedling

Skin needling, also known as collagen induction therapy or needle dermabrasion, is a relatively new technique [13,14]. It entails micropuncturing the skin from the papillary to the mid-dermis using a rolling instrument made up of several tiny, sharp needles [5]. In this procedure, the needling device is rolled back and forth with some pressure in different directions in the area of acne scars. Depending on their length, the needles can pierce the dermis between one and two millimeters [1,2]. It causes multiple microbruises in the dermis, which set off a chain reaction of growth factors that ultimately produce new collagen. It usually takes six to eight weeks for the results to become apparent. The best results, however, may not be seen for at least three months. The majority of patients need three to four treatments, spaced one month apart. Skin needling can be safely applied to all skin types because it has a lower risk of PIH than other procedures like dermabrasion, chemical peels, and laser resurfacing. It takes much less time to recover from than other resurfacing procedures. Also, it is less expensive than other resurfacing techniques. It is not advised for patients who have had collagen/filler injections, active skin infections, or a history of hypertrophic and keloidal scars within the last six months [2,3,5].

Soft tissue augmentation (dermal fillers)

Scars that are superficial and atrophic, like rolling and boxcar scars, can be effectively treated with soft tissue augmentation techniques [19]. Also known as dermal fillers, they restore the volume of soft tissue lost due to aging and promote the synthesis of collagen, which can further enhance the skin's natural texture. Injectable collagens used previously have become less popular as non-collagen fillers have emerged, with products containing hyaluronic acid (HA), poly-L-lactic acid (PLLA), and calcium hydroxyapatite (CaHA) taking their place. To some extent, all of these products can help improve the appearance of acne scars. Popularly referred to as dermal fillers, they can be broadly classified as short-lasting, semi-permanent, or long-lasting (Table 7) [2,12,23].

**Table 7:** Categorical overview of dermal fillers

Type of dermal filler	Estimated longevity	Common examples
Short-lasting (temporary)	6–18 months	Hyaluronic acid (HA)
Semipermanent	18–24 months	Poly-L-lactic acid (PLLA); calcium hydroxylapatite (CaHA)
Long-lasting (permanent)	>5 years	Polymethylmethacrylate (PMMA)

Short-Lasting Fillers

HA is a naturally occurring polysaccharide in the connective tissue. It is hydrophilic in nature and has low antigenic properties [12]. Hyaluronidase in the body naturally breaks down HA, and it is known to be a highly biodegradable substance. Innovative technological advancements have increased its clinical longevity to as much as 6-18 months by stabilizing HA through cross-linking [2]. By stimulating collagen synthesis and immediate water retention in the dermis, the injection of cross-linked hyaluronic acid restores the dermal matrix. When used in conjunction with subcision, HA fillers can enhance the appearance of atrophic acne scars. The product is injected beneath the skin that has been subcised to promote optimal wound healing. After one to three treatments, long-term results are visible [14].

Semi-permanent Fillers

CaHA is a semi-permanent dermal filler. It is bio-stimulatory in nature and made up of hydrogel-encased calcium hydroxyapatite microspheres. The microspheres stimulate the synthesis of collagen, which grows over the scaffold of calcium hydroxyapatite. While the hydrogel part corrects volume instantly, it gradually deteriorates. Because of the gel's special capacity to mold, uneven contours can be avoided. Although deeper ice-pick scars are not amenable to calcium hydroxyapatite treatment, shallow, atrophic acne scars can be improved. A single injection can improve appearance, and the effects last for a year. If required, treatment can be repeated one month after the first injection [12,14].

PLLA is a synthetic, biodegradable, long-lasting, and bio-stimulatory dermal filler. It stimulates collagen production, which grows around the PLLA deposited in the tissue, in a manner akin to CaHA action. In contrast to HA, it takes a few weeks to months for optimal collagen production and ECM remodeling to occur. Results generally last around two years, and patients may frequently need several injections of PLLA spaced one month apart to see the greatest improvement. PLLA produces limited correction for ice pick scars. Furthermore, PLLA carries a risk of nodule formation, erythema, bruises, and pain, particularly if applied in the incorrect dermal plane [2,12,27].

Permanent Fillers

Polymethylmethacrylate (PMMA) is a synthetic non-biodegradable polymer. It is composed of PMMA suspended in lidocaine and bovine collagen. Studies have reported improvement in the majority of patients using PMMA filler for the treatment of acne scarring. Temporary bruises, ecchymosis, erythema, and injection site pain were reported in these studies. Long-term studies also have reported granuloma formation with PMMA filler [2,14].

Platelet-rich plasma

Platelet-rich plasma (PRP) is a platelet concentrate derived from a person's own blood. It releases a variety of growth factors and cytokines at high concentrations from the platelets upon its injection into the dermis, stimulating the healing of wounds [9]. PRP is a new therapeutic option in dermatology that has been applied to a number of medical conditions, such as tendinitis, osteoarthritis, alopecia, melasma, and skin rejuvenation. A few studies that have been done using PRP treatment for acne scars have demonstrated that it is a safe procedure that can produce mild to moderate clinical improvement. Studies done using PRP as an adjunct to microneedling or TCA-CROSS or laser procedures reported superior outcomes compared to these procedures when done without PRP [2,9,23].

Stem cell

Stem cell therapy for acne scars is an emerging modality that leverages the regenerative potential of stem cells to improve acne scarring. The procedure typically involves harvesting a person's own stem cells, often from adipose (fat) tissue or bone marrow, although there are also stem cell-based products available. These stem cells are then processed and injected into the scarred areas or applied topically [2]. The stem cells work to promote tissue repair and regeneration by stimulating the production of collagen, elastin, and other essential components of healthy skin. A study conducted in Korea reported significantly higher improvement in acne scars with adipose tissue-derived stem cell exosomes and fractional laser compared to the control sides. While research is ongoing, early results suggest that stem cell therapy can lead to visible improvements in scar texture, color, and overall appearance. It's considered a minimally invasive and safe procedure, with minimal downtime [2,28].

Radiofrequency

Radiofrequency (RF) is another developing instrument that is commonly applied to skin rejuvenation [9]. It creates an electric current by heating the dermis with electromagnetic radiation, which causes collagen synthesis and skin contraction, thereby softening the scar [23]. RF is a non-ablative form of treatment and can be used alone or in combination with laser resurfacing methods. Traditional unipolar or monopolar RF uses a single electrode to enable deep dermal penetration; however, this approach is more painful and uncomfortable. Recent developments in RF, like bipolar RF, have made it possible to precisely deliver RF energy to deeper tissues while causing less damage to the epidermis above. Various types of radiofrequency devices used in acne scar treatment are described in Table 8 [24]. Using a variety of electrodes, fractional RF promotes dermal remodeling by creating a thermal wound. Microneedle RF is now widely used for scar treatment because it allows precise control over the depth at which the tissue is heated using microneedles. Acne scars like ice pick and boxcar scars give good results when treated with microneedle bipolar RF and fractional bipolar RF. The best results are observed after three to four treatment sessions and three months following the last treatment [24]. Compared to ablative lasers, RF has less downtime, infection risk, and scarring risk. It can be safely used on any type of skin. Common side effects of RF include erythema, scabbing, and temporary pain that subsides within three to five days [23,24].

**Table 8:** Types of radiofrequency devices used to treat acne scars RF, radiofrequency.

Radiofrequency devices	Description
Monopolar RF	Two electrodes with one in contact with the skin and the other as the grounding pad to target deep dermis
Bipolar RF	Two active electrodes that are placed a short distance apart from the intended treatment area to create more focused energy delivery and be less painful than monopolar RF
Fractional bipolar RF	Creates fractional zones of electrothermal damage in the dermis using electrodes or microneedles inserted into the skin

Lasers

Among the most widely used treatments for acne scars are laser treatments. They are divided into two primary categories: non-ablative (which can be traditional or fractional) and ablative (traditional or fractional) [23,24]. Traditional ablative lasers cause epidermal and dermal destruction leading to good clinical outcomes; however, this comes with significant discomfort after the procedure, a protracted recovery period, and a risk of side effects [24]. In contrast, the less invasive non-ablative lasers target the dermis while largely sparing the epidermis, resulting in quicker recovery times and more tolerability. The process of fractional laser resurfacing involves directing energy into microscopic columns of dermal and epidermal tissue. There are two types of fractional lasers: ablative fractional laser (AFL) and non-ablative fractional laser (NAFL) [23,24].

**Table 9:** Laser modalities for acne scar *Not a laser. PDL, pulsed dye laser; KTP: potassium titanyl phosphate.

Traditional ablative	Traditional non-ablative	Fractional non-ablative	Fractional ablative
Ablative 10,600-nm CO_2_	1320-nm Nd:YAG	Fractional 1550-nm Er-doped	Fractional 10,600-nm CO_2_
Ablative 2940-nm Er:YAG	1064-nm Nd:YAG	Fractional 1540-nm Er:glass	Fractional 2940-nm Er:YAG
	1450-nm Nd:YAG		Fractional 2790-nm YSGG
	755-nm picosecond		
	585-/595-nm PDL		
	532-nm KTP		
	Intense pulse light*		

Traditional Ablative Lasers

When it comes to treating acne scarring, ablative lasers are thought to be the best option because they tighten the skin, contract collagen, and improve scar appearance significantly [24]. With non-ablative lasers, several sessions are necessary before significant results are observed; with them, one treatment session is sufficient. Compared to non-ablative lasers, ablative lasers are typically linked to a greater risk of dyspigmentation, scarring, infections, and prolonged healing during the procedure [23,24]. In separate studies done using high-energy pulsed CO_2_ and long-pulsed Er:YAG lasers for acne scars, the reported clinical improvement was significant in about 75% of study populations [23,24]. However, ablative lasers are becoming a less common option for treating acne scars due to their lengthy recovery times and adverse effects [2].

Traditional Non-ablative Lasers

These are more widely used lasers, such as the 1064-nm and 1320-nm Nd:YAG as well as the 585/595-295-nm pulsed dye laser (PDL), and are noninvasive [24]. Non-ablative lasers target the dermis through a process called selective photothermolysis, in which the dermis receives photothermal energy without the surrounding epidermis being destroyed. This reduces downtime following the procedure and reduces damage to the epidermis. Rolling scars and shallow boxcar scars yield the best results [2]. Icepick scars benefit less from them. These lasers typically produce mild to moderate clinical results, necessitating several treatment sessions [23,24].

PDL is considered the gold standard for managing scar-associated erythema (SAE) [24]. It improves the clinical appearance of erythema by using selective thermolysis to reduce dermal vascular components. Treatments for SAE were also successfully done using another laser, potassium titanyl phosphate (KTP, or frequency-doubled Nd:YAG) [24]. Another treatment that has been found to be beneficial for SAE is intense pulsed light (IPL), a light source that emits incoherent light at a range of wavelengths (500-1,200 nm). Overall, due to their superior tolerance and quicker recovery time compared to ablative lasers, non-ablative lasers are becoming more and more common for treating acne scarring [24].

Ablative Fractional Lasers (AFLs)

AFLs combine the effectiveness of ablative lasers with the less severe side effect profile of fractional lasers. However, in order to produce a noticeable clinical improvement, several treatments are frequently needed. Common side effects from AFL include erythema, post-inflammatory hyperpigmentation (PIH), and discomfort during the procedure, which usually resolves within a few days or up to a month [23,24].

Non-ablative Fractional Lasers (NAFLs)

Compared to AFLs, NAFLs require multiple treatments but have less adverse effects and shorter downtime [23]. Rolling and boxcar scars respond well to NAFLs. However, there is a chance of developing PIH in patients with darker skin types, similar to AFLs. Moreover, the risk of PIH in patients with Fitzpatrick skin types IV to VI can be reduced by lowering the total treatment densities, reducing the number of passes, and applying topical depigmenting treatment both before and after treatment. Studies have also demonstrated that the level of outcome is usually correlated with the number of NAFL treatment sessions [23,24].

Emerging Laser Technologies

One of the newest technologies developed for treating acne scars is the picosecond 755 nm Alexandrite laser. It emits ultrashort pulses in the picosecond range, allowing for precise targeting of acne scars. It is also well-suited for addressing pigmentation commonly associated with acne scarring. During treatment, the Picosecond Alexandrite laser breaks down pigment particles within the scar tissue, promoting new collagen production and skin rejuvenation. This results in a reduction of pigmentation, textural irregularities, and overall scar visibility. Furthermore, the ultrafast pulses lessen heat-related tissue damage, resulting in faster recovery times and a lower chance of complications. Compared to traditional lasers, a noticeable improvement in scar appearance is seen with fewer treatment sessions [24].

Practical considerations of acne scar management

Treatment of acne scars should always have an algorithmic approach, which targets each component of scarring. After the complete evaluation of the patient, first treat the scar-associated erythema (SAE) if present, then consider various treatment modalities for atrophic scarring. The strategy for atrophic scarring treatment will depend on the types of scars that are present or predominate (Tables 10, 11). The greatest chance of a noticeable improvement can be obtained by customizing combination treatment for each patient. The best strategy to avoid or minimize acne-related scarring is still to treat active acne as soon as possible. It is also essential to treat active acne before approaching actual scar treatment so as not to create a sequence of events where active lesions continue to scar in areas that have already received scar treatment [14,23,24,29].

**Table 10:** Acne scarring treatment approach PDL, pulsed dye laser; KTP, potassium titanyl phosphate; IPL, intense pulse light; PRP, platelet-rich plasma; CROSS, chemical reconstruction of skin scarring.

Acne scar subtypes	Erythematous	Ice pick	Boxcar	Rolling
Generalized treatment	PDL	Lasers: ablative and non-ablative	Lasers: ablative and non-ablative	Lasers: ablative and non-ablative
	KTP	Microneedling +/- PRP	Microneedling +/- PRP	Microneedling +/- PRP
	IPL	Radiofrequency: microneedle or fractional bipolar	Radiofrequency: microneedle or fractional bipolar	Radiofrequency: microneedle or fractional bipolar
	Adjunctive topical peptides	Adjunctive topical peptides	If shallow: dermabrasion, chemical peel	Dermabrasion
			Adjunctive topical peptides	Chemical peel
				Adjunctive topical peptides
Individual treatment		Punch excision	Punch excision/elevation	Injectable filler
		CROSS technique	Injectable filler	Subscision
			If narrow: CROSS technique	

**Table 11:** Clinical efficacy of acne scar treatments as per atrophic scar subtypes +++, Good; ++, moderate; +, mild; -, no role/evidence, TCA-CROSS, trichloroacetic acid-chemical reconstruction of skin scarring.

Scar type	Ice pick	Deep boxcar	Shallow boxcar	Rolling
Chemical peel	+++ (TCA-CROSS)	++	++	++
Dermabrasion	+	+	++	+++
Microdermabrasion	+	+	++	+
Subcision	+	++	++	+++
Microneedling	+	+	++	++
Punch excision	+++	+++	+++	-
Punch elevation	-	++	+++	-
Filler	+	++	++	++
Radiofrequency	+	+++	++	++
Ablative fractional laser	+	+++	++	+++
Non-ablative fractional laser	+	++	++	++

## Conclusions

Acne scarring is an unintended but common complication in people who have suffered from acne. It significantly affects the quality of life. Though there are a multitude of treatment options available for acne scars, there is always a search for the ideal treatment approach for the best results. Moreover, continuous scientific research is ongoing for improving acne scars with multiple newer, innovative, and effective modalities as well as approaches. To achieve the best outcomes, the synergy of multiple treatment modalities is a must. While choosing any treatment(s), it is also important to remember that each scar reduction modality has advantages and disadvantages, and the selection of each treatment modality should be as per the individual patient's need and based on its clinical efficacy and safety.
